# Admission ECG-derived resting heart rate and in-hospital mortality after acute stroke: a multicenter retrospective cohort study

**DOI:** 10.3389/fmed.2026.1845558

**Published:** 2026-07-14

**Authors:** Salahaldeen Deeb, Alhareth M. Amro, Anas Ishqair, Ahmad Nasereddin, Mohammad Ishqair, Mohanad Samaheen, Naser Amro, Abdelwadod Abuturki, Sharif Basal

**Affiliations:** 1 Faculty of Medicine, Al-Quds University, Jerusalem, Palestine; 2 Department of Internal Medicine, Al-Ahli Hospital, Hebron, Palestine; 3 Interventional Neuroradiology Unit, Al-Ahli Hospital, Hebron, Palestine

**Keywords:** acute stroke, atrial fibrillation, electrocardiography, in-hospital mortality, multicenter cohort, resting heart rate

## Abstract

**Background:**

Early triage after acute stroke requires markers that are immediately available and clinically interpretable. The prognostic value of a single admission electrocardiogram (ECG)-derived resting heart rate (RHR), especially by atrial fibrillation (AF) status, remains insufficiently described.

**Methods:**

We conducted a multicenter retrospective cohort study of adults hospitalized with ischemic stroke, hemorrhagic stroke, or transient ischemic attack at five tertiary hospitals in Palestine between January 2018 and January 2025. The exposure was the first available 12-lead ECG-derived RHR recorded at rest. The primary outcome was in-hospital all-cause mortality. Multivariable logistic regression adjusted for age, sex, hypertension, diabetes mellitus, ischemic heart disease, smoking, prior stroke, AF, and stroke subtype.

**Results:**

The analytical cohort included 2,247 index hospitalizations (mean age 68.4 ± 13.6 years; 57.3% male), and 177 deaths occurred during hospitalization (7.9%). AF was present in 315 admissions (14.0%). Each 10-beats/min increase in admission RHR was associated with 12% higher odds of in-hospital death (adjusted odds ratio 1.12, 95% confidence interval 1.06–1.20; *p* < 0.001). Descriptively, mortality was higher with RHR ≥ 76 beats/min than with lower RHR (10.9% vs. 4.8%). In rhythm-stratified descriptive analyses, this separation was clearer without AF (9.0% vs. 3.6%; crude odds ratio 2.65, 95% confidence interval 1.78–3.96) than with AF (20.0% vs. 15.0%; crude odds ratio 1.42, 95% confidence interval 0.77–2.61).

**Conclusion:**

Admission ECG-derived RHR was associated with all-cause in-hospital mortality in this multicenter acute-stroke cohort. Because standardized stroke-severity measures, exact ECG timing, heart-rate-modifying medications, and cause-specific mortality were not uniformly available, this association should be interpreted as pragmatic early risk information rather than evidence that RHR is an independent causal determinant or therapeutic target.

## Introduction

Stroke remains a leading cause of death and long-term disability worldwide, and the early identification of patients at increased risk for adverse in-hospital outcomes remains a central objective of acute stroke care (1). In the first hours of admission, clinicians need variables that are available immediately, require no specialized processing, and can support decisions about monitoring intensity, escalation of care, and the urgency of multidisciplinary intervention. From that perspective, bedside and admission physiologic markers remain clinically attractive because they can be captured before more elaborate laboratory or imaging-derived prognostic information becomes available.

Resting heart rate (RHR) is one of the simplest physiologic variables available at admission, yet it integrates multiple biologically relevant processes, including autonomic tone, cardiovascular reserve, systemic stress, and hemodynamic compensation (2, 3). In acute cerebrovascular disease, central autonomic dysfunction and sympathetic activation may amplify the relevance of heart-rate abnormalities by increasing myocardial oxygen demand, promoting arrhythmogenesis, altering vascular tone, and potentially worsening already vulnerable cerebral perfusion (3, 4). For this reason, admission heart rate may plausibly function as an early marker of acute physiologic stress after stroke rather than merely as a descriptive vital sign.

Existing literature supports an association between higher heart rate and worse outcomes after stroke, but the structure of that evidence has important limitations for bedside interpretation. Prior studies have frequently focused on ischemic stroke alone, used repeated heart-rate measurements over the first 24–72 h of admission, or examined longer-term functional and cardiovascular outcomes rather than in-hospital mortality (5, 6). Those studies are informative, but they do not fully answer the practical question clinicians face at the time of admission: whether the first routinely obtained ECG-derived resting rate already contains prognostic information before serial measurements accrue. In addition, atrial fibrillation (AF) complicates the interpretation of a single rate measurement because an irregular ventricular response may make one ECG-derived value less representative of the patient’s average rate burden (7, 8). Broader AF literature also supports considering rhythm status when interpreting heart-rate findings (8, 9).

Other ECG-derived markers may also provide prognostic information after stroke. Usalp and Bağırtan recently reported that the frontal QRS-T angle, calculated from the absolute difference between the automatically reported QRS and T-wave axes on resting ECG, was associated with mortality among patients with ischemic stroke in sinus rhythm (10). That study supports the broader concept that routinely available ECG parameters may capture cardiac electrical or autonomic vulnerability after cerebrovascular events. However, their analysis focused on ischemic stroke and longer-term mortality, whereas the present study evaluates admission ECG-derived RHR and all-cause in-hospital mortality across a broader acute-stroke admission spectrum.

The present study was therefore designed as a pragmatic multicenter cohort analysis of adults hospitalized across the acute stroke spectrum in Palestine. Our primary objective was to evaluate the association between admission ECG-derived RHR and in-hospital all-cause mortality during the index hospitalization. A secondary objective was to examine whether the descriptive relationship between higher and lower admission RHR differed according to AF status. We hypothesized that higher admission RHR would be associated with greater in-hospital mortality, and that the signal would be clearer in patients without AF than in those with AF.

## Materials and methods

### Study design and reporting

This retrospective multicenter observational cohort study was reported with attention to the Strengthening the Reporting of Observational Studies in Epidemiology (STROBE) framework (11). The study was conducted across five tertiary hospitals in Palestine and included consecutive adult hospitalizations for the acute stroke spectrum between January 2018 and January 2025. The unit of analysis was the index hospitalization, and the primary analytic aim was etiologic/prognostic association testing rather than formal clinical prediction-model development.

### Study population and eligibility criteria

Eligible records were hospitalizations of adults aged 18 years or older with ischemic stroke, hemorrhagic stroke, or transient ischemic attack (TIA) diagnosed during the index admission. Stroke subtype classification was based on routine clinical and neuroimaging documentation in the medical record at the participating centers. Hospitalizations were excluded if admission occurred more than 7 days after symptom onset, if the primary exposure or discharge status was unavailable, or if prespecified covariates required for multivariable analysis were missing. Because the study addressed an in-hospital endpoint, no post-discharge follow-up period was incorporated into the primary analysis.

Data availability was assessed across the five participating hospitals before model specification. Variables were included in the multivariable analysis only when they were recorded with sufficient consistency in routine records across centers. Standardized baseline neurologic severity measures, including National Institutes of Health Stroke Scale score and Glasgow Coma Scale, as well as infarct volume, large-vessel-occlusion status, detailed lesion location, and time-stamped use of intravenous thrombolysis or mechanical thrombectomy, were not uniformly available in the harmonized dataset. These variables were therefore not included in the prespecified adjusted models, and their absence is treated as an important limitation rather than evidence that they were unimportant.

### Exposure definition

The exposure of interest was admission ECG-derived RHR, defined as the heart rate automatically reported on the first available 12-lead ECG obtained with the patient at rest during the index hospitalization. In routine practice at the participating hospitals, this ECG was generally obtained during the emergency department or admission-area evaluation after arrival and initial clinical assessment. However, exact clock time from hospital arrival to ECG acquisition, whether the ECG preceded or followed thrombolysis, and whether it occurred before ICU transfer or after clinical stabilization were not recorded consistently across centers. Accordingly, “admission ECG” in this study should be interpreted as the first routinely available in-hospital resting ECG rather than a uniformly pre-thrombolysis, pre-ICU, or post-stabilization measurement. The inferential emphasis of the study was placed on a continuous specification of RHR, modeled per 10-beats/min increase, in order to preserve information and reduce dependence on arbitrary categorization (12). A binary higher-versus-lower RHR classification using the cohort median, ≥ 76 versus < 76 beats/min, was retained only for descriptive presentation and bedside interpretability; it was not treated as a clinically validated threshold or therapeutic target.

### Outcomes and covariates

The primary outcome was in-hospital all-cause mortality during the index hospitalization, defined as death occurring between admission and discharge. Length of stay was calculated from recorded admission and discharge dates and is reported to contextualize the time window over which the primary outcome occurred. Intensive care unit (ICU) admission was treated as a secondary in-hospital descriptive outcome and is reported for clinical context, but it was not used as an adjustment variable because it occurs after exposure ascertainment and may lie on the causal pathway between early physiologic instability and death.

Prespecified covariates were age, sex, hypertension, diabetes mellitus, ischemic heart disease, smoking status, prior stroke, AF status, and stroke subtype. Stroke subtype was retained in all adjusted models because ischemic stroke, hemorrhagic stroke, and TIA differ substantially in baseline mortality risk and clinical trajectory. Data on chronic and acute medications that may influence heart rate, including beta blockers, non-dihydropyridine calcium channel blockers, antiarrhythmic agents, vasopressors, sedatives, analgesics, and other rate-modifying therapies, were not available with sufficient completeness and timing consistency for multivariable adjustment. Cause-specific mortality was also not uniformly adjudicated in the medical records; therefore, the primary endpoint was limited to all-cause in-hospital mortality to avoid differential misclassification of mechanism-specific deaths.

### Statistical analysis

Continuous variables are presented as mean ± standard deviation (SD) or median (interquartile range [IQR]) as appropriate, and categorical variables are summarized as counts and percentages. Group comparisons used the chi-square test for categorical variables and the Student t test or Mann-Whitney U test for continuous variables, depending on distributional assumptions.

Multivariable logistic regression was used to estimate odds ratios (ORs) and 95% confidence intervals (CIs) for in-hospital mortality. A base clinical model included age, sex, hypertension, diabetes mellitus, ischemic heart disease, smoking, AF, prior stroke, and stroke subtype. The primary heart-rate analysis entered admission RHR as a continuous variable per 10-beats/min increase in a separate adjusted model using the same covariate set. The binary median-derived RHR grouping was retained for descriptive comparison of mortality proportions and crude odds ratios rather than as the primary inferential parameterization. Prespecified rhythm-stratified analyses compared mortality across the higher and lower RHR groups in patients with and without AF. An exploratory unadjusted interaction test between AF status and the binary RHR category was also performed to assess directional heterogeneity.

The primary model combined ischemic stroke, hemorrhagic stroke, and TIA because the clinical question was whether a universally available admission ECG parameter carried early prognostic information across the pragmatic acute-stroke admission spectrum encountered at first hospital evaluation. To address the known heterogeneity in pathophysiology and baseline mortality risk, stroke subtype was included *a priori* in all adjusted models. *Post-hoc* sensitivity analyses were considered to evaluate the influence of stroke subtype, including analyses excluding TIA and analyses restricted to ischemic and hemorrhagic stroke where event numbers permitted. TIA-specific regression was not fitted because only three in-hospital deaths occurred among TIA admissions.

Because the goal of the study was to quantify association rather than build or validate a bedside prediction rule, receiver operating characteristic curves, area under the curve, sensitivity, and specificity were not primary analytic targets. All tests were two-sided, and *p* < 0.05 was considered statistically significant.

### Ethics approval

The study protocol was approved by the Research Ethics Committee of Al-Quds University, the relevant national ethics authorities, and the institutional review boards of the participating hospitals. Because the study used retrospectively collected, de-identified routinely collected clinical data, the requirement for individual informed consent was waived.

## Results

### Cohort characteristics

The analytical cohort comprised 2,247 index hospitalizations for acute ischemic stroke, hemorrhagic stroke, or TIA. The mean age was 68.4 ± 13.6 years, and 1,287 admissions (57.3%) involved male patients. The median length of stay was 3 days (IQR, 1–6), underscoring that the primary endpoint represented a short-term in-hospital outcome rather than a prolonged follow-up interval. Overall, 177 in-hospital deaths occurred (7.9%), and 738 admissions (32.8%) required ICU care.

AF was documented in 315 hospitalizations (14.0%), whereas 1,932 hospitalizations (86.0%) occurred in patients without AF (Table 1). Patients with AF were more frequently represented in the higher-RHR category than patients without AF (61.9% vs. 48.2%, *p* < 0.001). AF was also more common in women than in men and more common in ischemic stroke than in hemorrhagic stroke or TIA. These findings support the clinical plausibility of handling rhythm status as an important contextual variable when interpreting a single admission heart-rate measurement.

**TABLE 1:** Baseline clinical profile according to admission rhythm status and heart-rate category distribution.

Characteristic	Total (*n* = 2247)	Without AF (*n* = 1932)	With AF (*n* = 315)	*P*-value
Male sex, *n* (%)	1287 (57.3)	1136 (58.8)	151 (47.9)	< 0.001
Female sex, *n* (%)	960 (42.7)	796 (41.2)	164 (52.1)	
Hemorrhagic stroke, *n* (%)	423 (18.8)	403 (20.9)	20 (6.3)	< 0.001
Ischemic stroke, *n* (%)	1560 (69.4)	1304 (67.5)	256 (81.3)	
TIA, *n* (%)	264 (11.7)	225 (11.6)	39 (12.4)	
RHR < 76 bpm, *n* (%)	1120 (49.8)	1000 (51.8)	120 (38.1)	< 0.001
RHR ≥ 76 bpm, *n* (%)	1127 (50.2)	932 (48.2)	195 (61.9)	

AF indicates atrial fibrillation; RHR, resting heart rate; TIA, transient ischemic attack.

### Characteristics according to in-hospital outcome

Characteristics stratified by in-hospital outcome are shown in Table 2. Compared with survivors, non-survivors were older (77.2 ± 10.4 vs. 67.6 ± 13.6 years, *p* < 0.001) and were less likely to be male (47.5% vs. 58.1%, *p* = 0.008). Hypertension was more common among non-survivors (84.7% vs. 76.6%, *p* = 0.019), whereas diabetes mellitus and ischemic heart disease did not differ significantly between outcome groups. Smoking was markedly more frequent among non-survivors (59.9% vs. 34.0%, *p* < 0.001), as were hemorrhagic stroke (30.5% vs. 17.8%, *p* < 0.001) and ICU admission (76.3% vs. 29.1%, *p* < 0.001).

**TABLE 2:** Clinical characteristics according to in-hospital outcome.

Variable	Total (*n* = 2247)	Alive (*n* = 2070)	Died (*n* = 177)	*P*-value
Age, mean ± SD (years)	68.4 ± 13.6	67.6 ± 13.6	77.2 ± 10.4	< 0.001
Male sex, *n* (%)	1287 (57.3)	1203 (58.1)	84 (47.5)	0.008
Female sex, *n* (%)	960 (42.7)	867 (41.9)	93 (52.5)	
Hypertension, *n* (%)	1735 (77.2)	1585 (76.6)	150 (84.7)	0.019
Diabetes mellitus, *n* (%)	1188 (52.9)	1098 (53.0)	90 (50.8)	0.629
Ischemic heart disease, *n* (%)	138 (6.1)	132 (6.4)	6 (3.4)	0.154
Smoking, *n* (%)	809 (36.0)	703 (34.0)	106 (59.9)	< 0.001
History of stroke, *n* (%)	444 (19.8)	405 (19.6)	39 (22.0)	0.488
Hemorrhagic stroke, *n* (%)	423 (18.8)	369 (17.8)	54 (30.5)	< 0.001
Ischemic stroke, *n* (%)	1560 (69.4)	1440 (69.6)	120 (67.8)	
TIA, *n* (%)	264 (11.7)	261 (12.6)	3 (1.7)	
ICU admission, *n* (%)	738 (32.8)	603 (29.1)	135 (76.3)	< 0.001
Length of stay, median (IQR), days	3 (1–6)	3 (1–6)	6 (2–13)	< 0.001
RHR < 76 bpm, *n* (%)	1120 (49.8)	1066 (51.5)	54 (30.5)	< 0.001
RHR ≥ 76 bpm, *n* (%)	1127 (50.2)	1004 (48.5)	123 (69.5)	

ICU indicates intensive care unit; IQR, interquartile range; RHR, resting heart rate; TIA, transient ischemic attack.

Length of stay was also longer among non-survivors than survivors (median, 6 [IQR, 2–13] vs. 3 [IQR, 1–6] days; *p* < 0.001), which is consistent with a clinically unstable subgroup experiencing a more complicated hospital course. Admission RHR differed materially across outcome groups. In the higher-RHR category, in-hospital mortality was 10.9% (123/1,127) compared with 4.8% (54/1,120) in the lower-RHR category, an absolute difference of 6.1 percentage points. Put differently, 69.5% of all non-survivors belonged to the higher-RHR group, although that group accounted for only 50.2% of the overall cohort.

### Stroke subtype distributions

Stroke subtype distributions are detailed in Table 3. Ischemic stroke was the most frequent subtype (1,560 admissions, 69.4%), followed by hemorrhagic stroke (423, 18.8%) and TIA (264, 11.7%). Hemorrhagic stroke had the highest in-hospital mortality (12.8%) and the highest ICU utilization (50.4%), whereas TIA showed very low in-hospital mortality (1.1%). AF was more common in ischemic stroke (16.4%) than in hemorrhagic stroke (4.7%), and hypertension was especially prevalent in hemorrhagic stroke (89.1%). These descriptive differences reinforce the importance of retaining stroke subtype in the adjusted analyses to account for baseline differences in case severity and mortality risk.

**TABLE 3:** Patient characteristics by stroke subtype.

Variable	Total (*n* = 2247)	Hemorrhagic (*n* = 423)	Ischemic (*n* = 1560)	TIA (*n* = 264)	*P*-value
Age, mean ± SD (years)	68.4 ± 13.6	68.9 ± 12.9	68.5 ± 13.8	67.0 ± 13.6	0.200
Male sex, *n* (%)	1287 (57.3)	228 (53.9)	906 (58.1)	153 (58.0)	0.297
Female sex, *n* (%)	960 (42.7)	195 (46.1)	654 (41.9)	111 (42.0)	
Hypertension, *n* (%)	1735 (77.2)	377 (89.1)	1154 (74.0)	204 (77.3)	< 0.001
Diabetes mellitus, *n* (%)	1188 (52.9)	190 (44.9)	870 (55.8)	128 (48.5)	< 0.001
Ischemic heart disease, *n* (%)	138 (6.1)	24 (5.7)	99 (6.3)	15 (5.7)	0.831
Smoking, *n* (%)	809 (36.0)	190 (44.9)	560 (35.9)	59 (22.3)	< 0.001
Atrial fibrillation, *n* (%)	315 (14.0)	20 (4.7)	256 (16.4)	39 (14.8)	< 0.001
History of stroke, *n* (%)	444 (19.8)	87 (20.6)	306 (19.6)	51 (19.3)	0.893
ICU admission, *n* (%)	738 (32.8)	213 (50.4)	474 (30.4)	51 (19.3)	< 0.001
In-hospital death, *n* (%)	177 (7.9)	54 (12.8)	120 (7.7)	3 (1.1)	< 0.001

ICU indicates intensive care unit; TIA, transient ischemic attack. In-hospital deaths were rare among TIA admissions (3/264), so TIA-specific adjusted regression was not fitted.

As a descriptive sensitivity summary, exclusion of TIA left 1,983 ischemic or hemorrhagic stroke admissions and 174 in-hospital deaths, corresponding to 8.8% crude in-hospital mortality. This shows that the overall mortality endpoint was not numerically driven by TIA deaths, which were rare, with 3 deaths among 264 TIA admissions. However, because adjusted subtype-specific heart-rate models were not prespecified and the harmonized dataset did not support uniform adjustment for stroke severity, acute therapies, or heart-rate-modifying medication timing, these descriptive findings should not be interpreted as a substitute for subtype-specific adjusted analyses.

### Multivariable association between admission RHR and in-hospital mortality

Results of the multivariable models are shown in Table 4. In the base clinical model, older age remained strongly associated with in-hospital mortality (adjusted OR [aOR] 1.07 per year, 95% CI 1.06–1.09; *p* < 0.001). AF was independently associated with death (aOR 2.02, 95% CI 1.38–2.94; *p* < 0.01), as was prior stroke (aOR 2.09, 95% CI 1.29–3.38; *p* = 0.003). Relative to hemorrhagic stroke, both ischemic stroke and TIA were associated with lower in-hospital mortality. Male sex showed a borderline inverse association that did not meet conventional statistical significance (aOR 0.73, 95% CI 0.53–1.01; *p* = 0.057).

**TABLE 4:** Multivariable logistic regression for in-hospital mortality and adjusted continuous heart-rate effect.

Predictor	Adjusted OR (95% CI)	*P*-value
Age (per year)	1.07 (1.06–1.09)	< 0.001
Male sex	0.73 (0.53–1.01)	0.057
Hypertension	1.08 (0.74–1.59)	0.230
Diabetes mellitus	1.04 (0.64–1.71)	0.870
Ischemic heart disease	0.63 (0.26–1.54)	0.312
Smoking	1.05 (0.83–1.33)	0.350
Atrial fibrillation	2.02 (1.38–2.94)	< 0.01
History of stroke	2.09 (1.29–3.38)	0.003
Ischemic vs. hemorrhagic stroke	0.54 (0.38–0.78)	< 0.001
TIA vs hemorrhagic stroke	0.07 (0.02–0.24)	< 0.001
Admission RHR per 10-bpm increase*	1.12 (1.06–1.20)	< 0.001

*The heart-rate effect was estimated in a separate adjusted model using the same prespecified covariate set (age, sex, hypertension, diabetes mellitus, ischemic heart disease, smoking, atrial fibrillation, prior stroke, and stroke subtype). Standardized stroke-severity measures, exact ECG timing, heart-rate-modifying medications, acute reperfusion-therapy timing, and cause-specific mortality were not uniformly available for adjustment. The binary RHR split ( ≥ 76 vs. < 76 bpm) was retained for descriptive presentation rather than as the primary adjusted inferential parameterization. OR indicates odds ratio; CI, confidence interval; RHR, resting heart rate; TIA, transient ischemic attack.

In the primary heart-rate model, which used the same adjustment set but treated RHR as a continuous exposure, each 10-beats/min increase in admission RHR was associated with 12% higher odds of in-hospital death (aOR 1.12, 95% CI 1.06–1.20; *p* < 0.001). This graded estimate is the principal inferential heart-rate finding of the study because it avoids excessive information loss and does not imply a biologically privileged cut-point. The higher-versus-lower RHR split was therefore interpreted descriptively rather than as the primary adjusted exposure definition.

### Rhythm-stratified descriptive analyses

Descriptive rhythm-stratified comparisons are summarized in Table 5. Among patients without AF, mortality was 9.0% in the higher-RHR group and 3.6% in the lower-RHR group, corresponding to a crude OR of 2.65 (95% CI 1.78–3.96; *p* < 0.001). Among patients with AF, the corresponding mortality rates were 20.0% and 15.0%, respectively, corresponding to a crude OR of 1.42 (95% CI 0.77–2.61; *p* = 0.264). Although mortality remained numerically higher in the higher-RHR group even among patients with AF, the separation between groups was less distinct and substantially less precise than in the subgroup without AF. The exploratory unadjusted interaction test between AF status and the binary RHR category was directionally consistent with heterogeneity but did not cross the conventional threshold for statistical significance (*p* for interaction = 0.093).

**TABLE 5:** Descriptive rhythm-stratified association between admission heart-rate category and in-hospital mortality.

Group	Heart-rate category	*N*	Deaths, *n* (%)	Crude OR (95% CI)	*P*-value
Overall cohort	RHR < 76 bpm	1120	54 (4.8)	Reference	–
	RHR ≥ 76 bpm	1127	123 (10.9)	2.42 (1.74–3.37)	< 0.001
Without AF	RHR < 76 bpm	1000	36 (3.6)	Reference	–
	RHR ≥ 76 bpm	932	84 (9.0)	2.65 (1.78–3.96)	< 0.001
With AF	RHR < 76 bpm	120	18 (15.0)	Reference	–
	RHR ≥ 76 bpm	195	39 (20.0)	1.42 (0.77–2.61)	0.264

Binary higher-versus-lower heart-rate comparisons are presented descriptively. The primary inferential heart-rate estimate for the full cohort is the adjusted continuous model shown in Table 4. Exploratory unadjusted interaction between AF status and the binary RHR category: *p* for interaction = 0.093. AF indicates atrial fibrillation; OR, odds ratio; RHR, resting heart rate.

## Discussion

In this multicenter retrospective cohort study of adults hospitalized with acute stroke or TIA, admission ECG-derived RHR was associated with short-term in-hospital mortality. The central result of the study is the continuous adjusted estimate: every 10-beats/min increase in admission RHR was associated with 12% higher odds of death during the index hospitalization after adjustment for demographic characteristics, vascular comorbidities, AF, prior stroke, and stroke subtype. This finding supports the interpretation of admission heart rate as an integrated marker of early physiologic stress in acute cerebrovascular disease rather than as a purely descriptive vital sign.

Our findings are directionally consistent with prior reports showing that higher heart rate is associated with worse outcomes after stroke. Kuo et al. demonstrated that higher mean heart rate during the first 3 hospital days was associated with poorer 3-month functional outcome after acute ischemic stroke (5), and Lee et al. showed that acute-period heart-rate parameters were associated with 1-year outcomes after ischemic stroke (6). Lin et al. further linked higher resting heart rate after ischemic stroke to subsequent major adverse cardiovascular events (13), while You et al. reported that persistently high heart-rate trajectories and greater variability in acute intracerebral hemorrhage were associated with worse outcomes (14). Han et al. additionally highlighted that the prognostic interpretation of admission heart rate differs according to AF status in acute ischemic stroke (7). The present study extends that body of work in a pragmatic direction by focusing on the first routinely available ECG-derived resting rate, the index-hospitalization mortality window, and a broader acute-stroke spectrum from an underrepresented Middle Eastern setting.

The biological plausibility of the observed association is supported by the literature on the brain-heart axis after stroke (3, 4). Acute cerebral injury can disturb central autonomic pathways, particularly when insular, brainstem, or deep hemispheric networks are affected, leading to sympathetic overactivation and relative vagal withdrawal. In that context, tachycardia may reflect catecholamine excess, inflammatory stress, impaired cardiovascular reserve, or hemodynamic compensation. These processes may plausibly accompany early deterioration after stroke. Nonetheless, the observed association should not be interpreted as proof that lowering heart rate would reduce mortality, because RHR may be a marker of illness severity rather than a modifiable cause.

An important interpretive point is that the continuous adjusted model should carry greater weight than the binary higher-versus-lower comparison. Median-based thresholds are convenient for clinical exposition but may create an illusion of a discrete biological boundary where none exists (12). In the present study, the continuous analysis supported a graded association, whereas the binary split was retained only as a descriptive simplification. Accordingly, the finding should not be interpreted as identifying 76 beats/min as a treatment target or validated prognostic threshold.

The rhythm-specific findings also merit careful interpretation. In the descriptive subgroup comparisons, the separation between higher and lower RHR groups was clearer in patients without AF than in those with AF. This pattern is clinically plausible. In sinus rhythm, a single admission rate is more likely to reflect autonomic tone, systemic stress, and cardiovascular reserve. In AF, by contrast, one ECG-derived value may not capture average ventricular rate, rate burden, or rate variability over time (7, 9). The weaker signal in AF may therefore reflect measurement limitations as much as biological differences. It is also notable that AF itself remained associated with in-hospital mortality in the adjusted clinical model, indicating that rhythm status is prognostically relevant even when the interpretability of a single heart-rate measurement is reduced.

The study has practical clinical implications. This may be especially relevant in resource-constrained or referral-dependent health systems, where advanced imaging quantification, standardized severity documentation, and access to specialized acute interventions may be inconsistent across hospitals. In such settings, the value of ECG-derived RHR is not that it replaces neurologic assessment, but that it can provide a universally available and low-cost signal to supplement early bedside evaluation. A first admission ECG is nearly universal in contemporary stroke care, inexpensive, and rapidly interpretable. If its heart-rate component carries prognostic information, clinicians may be able to incorporate that signal into early bedside risk assessment before serial observations, advanced imaging review, or more comprehensive severity profiling are complete. At the same time, our findings do not support treating heart rate itself as a causal therapeutic target on the basis of this dataset alone. The current results are better understood as supporting admission RHR as a candidate early-risk marker rather than as a mandate for heart-rate-lowering intervention.

Several strengths should be noted. The study used a multicenter design, included the acute stroke spectrum rather than a single subtype alone, focused on an exposure that is available at the earliest point of care, and explicitly considered rhythm status when interpreting the exposure. The analytic emphasis on a continuous RHR specification also improves interpretability by reducing dependence on arbitrary categorization.

The limitations are important. First, the retrospective observational design precludes causal inference and leaves substantial potential for residual confounding. Most importantly, standardized stroke-severity variables, including NIHSS score, GCS, infarct volume, large-vessel-occlusion status, detailed lesion location, and consistently recorded use and timing of thrombolysis or mechanical thrombectomy were not uniformly available across centers. These factors may influence both admission RHR through acute stress or autonomic activation and the risk of in-hospital death. Second, the exposure was a single first available ECG-derived measurement rather than a standardized ECG obtained at a fixed time point. Exact timing relative to emergency department arrival, reperfusion therapy, ICU transfer, fever, pain, dehydration, respiratory distress, and clinical stabilization could not be consistently determined. Third, medication data were incomplete, especially for beta blockers, calcium channel blockers, antiarrhythmic agents, vasopressors, sedatives, analgesics, and other therapies that may alter heart rate. Fourth, cause-specific mortality was not reliably adjudicated, so deaths due to neurologic deterioration, arrhythmia, infection, respiratory failure, aspiration, or other causes could not be distinguished. Fifth, although stroke subtype was adjusted for, pooling ischemic stroke, hemorrhagic stroke, and TIA into one primary model may obscure subtype-specific associations; TIA in particular had very low mortality and should be considered separately in future analyses. Sixth, AF subgroup estimates were less precise because the subgroup was smaller and because a single ECG-derived rate is an imperfect representation of ventricular-rate burden in AF. Finally, the study was not designed as a formal prediction-model development project and does not provide calibration statistics, discrimination metrics, or clinically validated treatment thresholds.

In conclusion, admission ECG-derived RHR was associated with all-cause in-hospital mortality in this multicenter cohort of adults admitted with acute stroke or TIA. The primary continuous analysis supported a graded association between higher RHR and greater odds of death, and the descriptive binary comparison appeared more informative among patients without AF than among those with AF. However, because standardized stroke-severity variables, acute treatment details, heart-rate-modifying medication exposure, cause-specific mortality, and exact ECG timing were not uniformly available, the findings should be interpreted as prognostic and hypothesis-generating rather than causal. Prospective studies with standardized ECG timing, repeated rhythm-specific heart-rate measurements, subtype-specific analyses, and richer adjustment for neurologic severity and acute therapies are needed before admission RHR can be incorporated into clinical decision algorithms.

## Data Availability

The raw data supporting the conclusions of this article will be made available by the authors, without undue reservation.
